# Choreographing Well-Being: The Predictive Role of Self-Compassion on Life Satisfaction—A Therapeutic-Based Art Pedagogy Perspective in Recreational Dance

**DOI:** 10.3390/sports13070223

**Published:** 2025-07-08

**Authors:** Aglaia Zafeiroudi, Thomas Karagiorgos, Ioannis Tsartsapakis, Gerasimos V. Grivas, Charilaos Kouthouris, Dimitrios Goulimaris

**Affiliations:** 1 Department of Physical Education & Sport Sciences, University of Thessaly, 42100 Trikala, Greece; kouthouris@uth.gr; 2 Department of Physical Education & Sport Sciences, Aristotle University of Thessaloniki, 57001 Thessaloniki, Greece; thomaskg@phed.auth.gr; 3 Department of Physical Education & Sport Sciences, Aristotle University of Thessaloniki, 62122 Serres, Greece; ioantsar@phed-sr.auth.gr; 4 Physical Education and Sports, Division of Humanities and Political Sciences, Hellenic Naval Academy, Piraeus, 18539 Athens, Greece; grivasger@hotmail.com; 5 Department of Physical Education & Sport Sciences, Democritus University of Thrace, 69100 Komotini, Greece; dgoulima@phyed.duth.gr

**Keywords:** mindfulness in movement, performance psychology, dance education, creative embodiment, subjective well-being, expressive arts pedagogy

## Abstract

Dance encompasses physical, emotional, and social elements, creating a dynamic platform for the exploration of well-being. As a therapeutic approach, dance movement further applies these dimensions to enhance emotional resilience, foster mindfulness, and improve overall mental health. This study examined the relationship between self-compassion and life satisfaction among 912 recreational dancers (80% female and 20% male) in Greece. Participants completed the Self-Compassion Scale and Satisfaction with Life Scale. Confirmatory Factor Analysis validated the five-factor self-compassion model, and regression analysis identified predictors of life satisfaction. Self-kindness emerged as a strong positive predictor (β = 0.258, *p* < 0.001), while isolation (β = −0.307, *p* < 0.001) and self-judgment (β = −0.083, *p* = 0.029) negatively predicted life satisfaction. Common humanity (β = 0.064, *p* = 0.066) and mindfulness (β = 0.004, *p* = 0.907) showed no significant predictive effect. The model explained 21.7% of the variance in life satisfaction (R^2^ = 0.217). Small but statistically significant differences in self-compassion dimensions were observed across dance styles. Partner-oriented dancers such as those practicing tango reported slightly higher self-kindness and mindfulness, while ballet dancers showed a small increase in self-judgment and isolation. Life satisfaction remained consistent across styles, highlighting dance’s overall contribution to well-being. These findings suggest that integrating self-compassion training into dance education and psychotherapy, particularly within a Therapeutic-Based Art Pedagogy framework, may contribute to emotional resilience, foster social connection, and promote mental health, positioning dance as a potentially transformative tool for holistic development.

## 1. Introduction

Therapy uses dance movement as a distinctive approach to mental health by harnessing the interconnectedness of physical movement and emotional expression [1]. Rooted in the mind–body connection, dance movement therapy supports practitioners in accessing and processing emotions, often bypassing the limitations of verbal communication [2]. Studies have indicated that dance movement significantly reduces symptoms of anxiety and depression by fostering relaxation, embodied cognition, self-awareness, and self-expression [3,4,5]. These studies also highlight its role in enhancing emotional regulation and social connectivity, crucial factors for overall mental well-being. Moreover, the use of movement as a medium for therapy has been shown to improve the emotional resilience of individuals facing trauma, offering a safe space to explore and integrate difficult experiences.

Beyond its psychotherapeutic applications, dance also functions as an educational environment that supports emotional and psychological growth. This dual role is encapsulated in the concept of Therapeutic-Based Art Pedagogy, a pedagogical model that integrates expressive arts with therapeutic purpose [6]. Rooted in embodied learning, this approach recognizes the body as a site of emotional expression and transformation, enabling participants to develop self-awareness, regulate affect, and build interpersonal connections through movement [6,7,8,9]. In recreational dance contexts, where well-being and enjoyment are prioritized over performance mastery, Therapeutic-Based Art Pedagogy provides a meaningful framework for cultivating psychological resilience and social engagement alongside artistic development.

This dynamic interaction of movement, emotion, and interpersonal expression forms the basis for what is described as “choreographing well-being”, a process through which well-being is cultivated via embodied learning, reflective practice, and relational engagement in dance [6,7,9]. Within the framework of Therapeutic-Based Art Pedagogy, this concept emphasizes the active shaping of emotional resilience and life satisfaction through creative movement and shared experience [6,9].

Dance movement in therapy also has significant relevance in addressing the challenges faced by practitioners in performance-oriented activities, such as dance, where mental health issues like anxiety, perfectionism, and isolation are crucial [1,10,11]. It aligns closely with self-compassion dimensions, such as self-kindness and common humanity, which may be important in mitigating harsh self-criticism and promoting emotional well-being [12]. Research has also mentioned that participants in group-based dance movement therapy programs report reduced feelings of isolation and enhanced life satisfaction, reinforcing the social and therapeutic benefits of shared movement in fostering holistic well-being.

Self-compassion, as defined by Neff [13], encompasses self-kindness, mindfulness, and a sense of common humanity, which counterbalance self-judgment and isolation. Research highlights a consistent positive association between self-compassion and well-being, including life satisfaction [14,15,16]. Life satisfaction, as a component of subjective well-being, is influenced by self-compassion’s ability to reduce self-criticism and foster emotional resilience [17,18]. Among self-compassion dimensions, self-kindness has emerged as the most prominent among the predictors of life satisfaction, underscoring how responses to personal struggles shape well-being [13,19,20].

The self-compassion model has been validated through structural frameworks, including Neff et al.’s [21] bifactor and five-factor models. These studies confirm the interrelated nature of positive dimensions and the necessity of distinguishing between positive and negative traits [22]. Cross-cultural research further supports the five-factor model’s stability, emphasizing its universal applicability [23,24]. Nevertheless, the higher-order structure of the scale remains debated. Marsh et al. [25] highlighted conceptual and statistical concerns in previous modeling attempts and proposed a more refined bifactor structure using Bayesian estimation techniques. Their findings support interpreting the Self-Compassion Scale not only through its six subscales but also in terms of two overarching dimensions, compassionate and uncompassionate self-responding, rather than relying solely on a single global score. Mindfulness, as a positive dimension, has been shown to buffer against isolation’s harmful effects, reducing emotional distress [26].

Self-kindness has been consistently linked to life satisfaction, helping individuals manage negative emotions and enhance happiness [16,27]. Conversely, isolation, a key negative predictor, significantly reduces life satisfaction by exacerbating emotional distress and disconnection [21,28]. Common humanity fosters resilience during adversity, mitigating loneliness and promoting satisfaction with life [13,16].

In dance, where physical and emotional demands are high, self-compassion plays a critical role in enhancing well-being. Dancers face pressures from performance evaluations, self-criticism, and isolation, often resulting in anxiety and reduced life satisfaction [29,30]. Self-compassion helps moderate these stressors by fostering emotional resilience and improving emotional regulation, as seen in both athletic and performing arts contexts [31,32,33].

Research has indicated a strong positive correlation between self-compassion and life satisfaction in dancers, with self-kindness and mindfulness being key contributors [12]. Self-compassion reduces burnout and dissatisfaction, while lower self-judgment is associated with higher satisfaction [34,35]. Negative factors like self-judgment and isolation significantly decreased dancers’ life satisfaction, underlining the importance of fostering self-compassion to address these challenges [12,36].

While professional dance has been extensively studied in relation to self-compassion and life satisfaction, there remains a significant gap in research concerning recreational dance. Both groups may experience emotional challenges and could benefit from fostering self-compassion, but the mechanisms at play and the degree to which self-compassion influences life satisfaction may differ.

While the earlier analyses broadly focused on psychological predictors of well-being across both dancers and non-dancers, the present study offers a more focused investigation by isolating a homogeneous sample of recreational dancers and applying advanced psychometric modeling to validate the self-compassion construct. This continuation aims to deepen understanding of the mechanisms through which self-compassion dimensions influence life satisfaction within dance-specific contexts and to inform more specialized psychological interventions for dance educators and therapists.

The aim of this study was to investigate the direct effects of self-compassion components on life satisfaction among recreational dancers. This study aimed to explore how individual dimensions of self-compassion (self-kindness, mindfulness, common humanity, self-judgment, and isolation) relate to dancers’ overall life satisfaction, with the hypothesis that certain self-compassion components would have stronger predictive power. More specifically, the present study aimed to (i) explore the validity of the Self-Compassion scale among recreational dancers, (ii) test self-compassion’s scale predictive validity on life satisfaction, and (iii) examine differences between dance styles in life satisfaction and the five dimensions of self-compassion.

The present study focused on extending Neff’s self-compassion model [13] by testing these relationships within recreational dance. Understanding the relationship between self-compassion and life satisfaction within the dance field can inform the development of targeted psychological strategies aimed at enhancing well-being and promoting long-term engagement in dance and other art-based practices during leisure time. Although the Self-Compassion Scale has previously been used in Greek adult populations [33] and among recreational dancers [12], its factorial structure and reliability have not been formally validated in this group. Given the embodied and affective nature of recreational dance, establishing the construct validity of the scale in this specific context is both methodologically important and a key contribution of the present study.

## 2. Materials and Methods

### 2.1. Process

Participants completed a structured written questionnaire under the direct supervision of researchers at dance schools or studios before their practice sessions. Prior to their participation, all respondents were informed that their responses would remain strictly anonymous. To ensure clarity and facilitate completion, the questionnaire was accompanied by detailed written instructions and supplemented with oral explanations provided by the researchers.

This study was conducted in professional settings where instruction was delivered by experienced and certified dance educators. Data were collected between October 2024 and April 2025. All participants provided informed consent prior to participation, in accordance with ethical guidelines. Ethical approval for this study was granted by the Internal Ethics Committee (IEC) of the Department of Physical Education and Sport Science (DPESS), University of Thessaly, Greece (University of Thessaly’s Code of Ethics, Protocol Number: 2402, 5 June 2024).

### 2.2. Participants

This study involved 912 recreational dancers recruited from various dance studios and clubs offering instruction in different dance styles across multiple urban centers in Greece. Of the initial sample, 77 participants were excluded due to incomplete responses. An a priori power analysis was conducted using G*Power (version 3.1.9.7; refs. [37,38] to estimate the minimum required sample size for the multiple linear regression model employed in this study. Assuming a medium effect size (f^2^ = 0.15), an alpha level of 0.05, statistical power of 0.95, and five predictor variables, the analysis indicated that a minimum of 138 participants would be necessary. The actual sample size of 912 exceeded this requirement, ensuring high power to detect medium-sized effects with confidence.

Participants were selected based on the following inclusion criteria: (a) active engagement in both individual and group dance practices, (b) absence of participation in other physical exercise systematically, and (c) absence of participation in mind–body activities, structured wellness programs, or positive psychology interventions during the year preceding study and throughout its duration. Individuals who failed to meet these eligibility criteria were excluded from participation.

Only participants with complete responses on the primary variables were retained. Cases with missing data were excluded listwise. No imputation or data replacement methods were applied. Table 1 presents the demographic characteristics of the sample. Most participants were female (80%). Most dancers practiced contemporary, traditional, ballet, or Latin styles, and half of them had been dancing for at least 8 years.

### 2.3. Instruments

Life satisfaction was assessed using the Satisfaction with Life Scale (SWLS) [39]. For this study, the validated Greek adaptation of the SWLS [40] was used. The SWLS is a widely recognized instrument for measuring subjective well-being, designed to capture individuals’ global cognitive appraisal of their overall life satisfaction. The scale consists of five items that evaluate key dimensions of life satisfaction, such as whether individuals perceive their life as being close to their ideal or whether they have achieved their desired goals (e.g., “Most of the time, my life is close to my ideal”, “So far, I have gotten the important things I want in life”). Responses are recorded on a 7-point Likert scale ranging from 1 (strongly disagree) to 7 (strongly agree).

The Self-Compassion Scale (SCS), originally developed by Neff [13] and later adapted into Greek by Mantzios et al. [41], consists of 26 items designed to assess the extent to which individuals exhibit self-compassion. The scale is structured within six distinct factors: (1) self-kindness (5 items; e.g., “I am tolerant of my flaws and inadequacies”), (2) Self-Criticism (5 items; e.g., “When I see aspects of myself that I don’t like, I get upset with myself”), (3) Common Humanity (4 items; e.g., “I try to see my failures as part of human nature”), (4) Isolation (4 items; e.g., “When I fail at something important to me, I feel alone in my failure”), (5) mindfulness (4 items; e.g., “When I feel sad, I try to approach my feelings with curiosity and honesty”), and (6) overidentification (4 items; e.g., “When something painful happens, I exaggerate the event by giving it disproportionate significance”). Participants rated each item on a 5-point Likert scale, indicating the frequency of these behaviors, ranging from 1 (almost never) to 5 (almost always). For the purposes of the present study, the Overidentification subscale was excluded following CFA results that indicated inadequate factor loadings. Therefore, a five-factor model was adopted to ensure better psychometric validity in this population.

Additionally, participants completed a set of questions regarding their social and demographic characteristics.

### 2.4. Data Analysis

All statistical analyses were conducted using SPSS version 26 and AMOS version 26. Descriptive statistics were first computed for all variables to assess means and standard deviations. To examine the factorial structure of the Self-Compassion Scale in recreational dancers, a Confirmatory Factor Analysis (CFA) was performed using AMOS, testing a five-factor model based on Neff’s theoretical framework. Confirmatory Factor Analysis was conducted using the Maximum Likelihood estimation method. Model fit was evaluated using the Comparative Fit Index (CFI), Root Mean Square Error of Approximation (RMSEA), the Standardized Root Mean Square Residual (SRMR), and the chi-square divided by degrees of freedom (χ^2^/df). Internal consistency was assessed using Cronbach’s alpha coefficients for each subscale of the Self-Compassion Scale. Pearson correlation coefficients were calculated to explore the bivariate relationships between self-compassion components and life satisfaction. To examine the predictive role of self-compassion components on life satisfaction, a multiple linear regression analysis was conducted. Standardized beta coefficients (β), structure coefficients (rs), and the Pratt Index were calculated to interpret the relative importance and contribution of each predictor. The adjusted R^2^ value was used to evaluate the model’s overall explanatory power. Additionally, a one-way Analysis of Variance (ANOVA) was performed to assess differences in self-compassion dimensions and life satisfaction across different dance styles. Scheffé post hoc tests were applied to correct for Type I error.

## 3. Results

### 3.1. Descriptive Statistics

The means and standard deviations of the variables examined in the present study are summarized in Table 2.

Normality of the data distribution was assessed by calculating skewness and kurtosis values for each variable. These results indicated that the data met the criteria for normal distribution, as the absolute skewness values did not exceed 2, and the absolute kurtosis values remained below 7, consistent with recommendations for large samples (*n* > 300; [42]).

### 3.2. Measurement Model

A Confirmatory Factor Analysis (CFA) was conducted using the Maximum Likelihood estimation method to examine the five-factor structure of the Self-Compassion Scale (SCS). The initial model included all 26 items from the original scale. Upon evaluating the standardized factor loadings and modification indices, the three items corresponding to the “Overidentification” subscale exhibited loadings below the 0.50 threshold [43], indicating poor representation of the latent factor. As a result, these items were removed to improve model fit and structural clarity. A revised model was subsequently tested with the remaining 23 items across five dimensions: self-kindness, self-judgment, common humanity, mindfulness, and isolation. The assessment of the measurement model revealed: *χ*^2^ (532,85)/*df* (145) = 3.1, *p* < 0.001, RMSEA = 0.09, SRMR = 0.06. CFI = 0.91 (Table 3), all meeting established thresholds for acceptable model fit [44].

Standardized factor loadings ranged from 0.47 to 0.71, all statistically significant (*p* < 0.001). The standardized factor loadings for each of the self-compassion dimensions are presented in Table 4.

Regarding reliability, Cronbach’s alpha indicators were above the recommended threshold of 0.75. Also, the Composite Reliability (CR) ranged between 0.71 and 0.78 [43]. Hence, the internal and construct reliability were established. Moreover, the Average Variance Extracted (AVE) values ranged from 0.53 to 0.61, which were above the 0.50 threshold, demonstrating satisfactory convergent validity for the measurement model [45]. The intercorrelations among the five latent factors of the Self-Compassion Scale are shown in Table 5, supporting the theoretical coherence of the multidimensional construct.

### 3.3. Regression Analysis

A standard multiple linear regression (Enter method) was conducted to examine the extent to which the five self-compassion components predicted life satisfaction. Prior to interpreting the results, assumptions of multicollinearity were assessed using collinearity diagnostics. All predictors showed Variance Inflation Factor (VIF) values below 2 and tolerance values above 0.50, indicating no evidence of multicollinearity. The analysis indicated that self-kindness positively predicted life satisfaction (β = 0.258, *p* < 0.001), and isolation (β = −0.307, *p* < 0.001) was a significant negative predictor of life satisfaction. Self-judgment was also negatively related to life satisfaction (β = −0.083, *p* = 0.029). Conversely, mindfulness showed no significant effect (β = 0.004, *p* = 0.907), and common humanity contributed less to life satisfaction (β = 0.064, *p* = 0.066). The overall model explained 21.7% of the variance in life satisfaction (*R*^2^ = 0.217, Adjusted *R*^2^ = 0.212). Structure coefficients and Pratt Index values further confirmed the relative importance of isolation (Pratt = 0.119) and self-kindness (Pratt = 0.099) as the strongest contributors to the model. These findings are summarized in Table 6.

The overall regression model was significant, *F*(5, 887) = 49.102, *p* < 0.001, explaining 21.7% of the variance in life satisfaction (adjusted R^2^ = 0.212), with a standard error of estimate of 0.918. The results suggest that self-kindness and isolation may play meaningful roles in predicting life satisfaction among dancers. Self-judgment was found to negatively impact life satisfaction, while common humanity contributed less to the model. Mindfulness did not show a significant effect.

### 3.4. Differences Between Dance Styles in Life Satisfaction and the Five Dimensions of Self-Compassion

A one-way ANOVA was performed to compare the effects of different dance styles on the five dimensions of self-compassion and life satisfaction. Descriptive statistics for each dependent variable by dance style are presented in Table 7.

The analysis revealed that there was a statistically significant difference in common humanity, (*F*(6, 905) = 2.872, *p* = 0.009, *η*^2^ = 0.019), self-kindness (*F*(6, 905) = 3.575, *p* = 0.002, *η*^2^ = 0.023), mindfulness (*F*(6, 905) = 4.951, *p* < 0.001, *η*^2^ = 0.032), and self-judgment (*F*(6, 905) = 3.522, *p* = 0.002, *η*^2^ = 0.023). The result showed no statistically significant differences in life satisfaction (*F*(6, 905) = 1.062, *p* = 0.384, *η*^2^ = 0.007) and isolation (*F*(6, 905) = 1.268, *p* = 0.270, *η*^2^ = 0.008). These effect sizes, although statistically significant in some cases, indicate small impacts of dance style on these psychological dimensions [46]. To control for the increased risk of Type I error, Scheffé post hoc tests were used.

Results revealed that Tango dancers reported significantly higher levels of common humanity and mindfulness compared to Ballet participants (*p* < 0.05), with medium effect sizes (*d* = 0.50). Tango dancers also scored significantly higher than participants in the Aerobic/Zumba group in mindfulness (*d* = 0.63), indicating a moderate-to-large effect. The results are presented in Table 8. Only statistically significant post hoc comparisons are presented below.

## 4. Discussion

An important methodological contribution of this study is the validation of the five-factor structure of the Self-Compassion Scale in a large sample of Greek recreational dancers. While the SCS had been previously used in this population, this is the first study to formally confirm its psychometric integrity through CFA. This validation supports the use of the SCS in embodied leisure contexts, such as recreational dance, and extends its applicability to cross-cultural and art-based populations. By providing psychometric evidence in a group characterized by physical expressiveness and emotional engagement, this study strengthens the methodological foundations of self-compassion research and contributes to its theoretical refinement.

This study provided supportive evidence that self-compassion, as a multidimensional construct, plays a crucial role in shaping life satisfaction among recreational dancers. This study confirmed the five-factor model of self-compassion, a finding consistent with prior research validating the model’s robustness across various contexts [21,22,24,47]. These results further support the reliability and relevance of the model across diverse populations.

Recent studies, such as Marsh et al. [25], have further discussed the factor structure of the Self-Compassion Scale, proposing bifactor models that distinguish between compassionate and uncompassionate self-responding as distinct global factors. While the present study adopted the five-factor model validated in prior research, this newer line of evidence highlights the ongoing theoretical and psychometric evolution of the construct. It supports the continued use of both total and subscale scores depending on the analytic focus, especially in applied settings such as dance.

Additionally, self-kindness and reduced isolation emerged as significant predictors of life satisfaction, while self-judgment negatively affected life satisfaction. Mindfulness, however, did not show a significant predictive relationship with life satisfaction among dancers. These findings contributed to the growing body of literature on self-compassion and psychological well-being, with implications for dancers and other art-based populations.

Although the Therapeutic-Based Art Pedagogy model was not directly tested in the present analyses, its inclusion provided a conceptual framework to contextualize the psychological relevance of dance as a reflective, embodied practice. This model emphasizes the integration of emotional expression, somatic awareness, and relational learning, elements present in many recreational dance settings. Referencing this approach aims to highlight the broader educational and therapeutic dimensions of dance that intersect with the psychological variables explored in the study, such as self-compassion and life satisfaction.

### 4.1. Self-Kindness as a Strong Predictor of Life Satisfaction

The finding that self-kindness significantly predicted life satisfaction aligns with existing literature on self-compassion, highlighting its potential role in enhancing well-being [48]. This result not only supports but also extends prior research, emphasizing self-kindness as a key adaptive element of self-compassion and a strong predictor of life satisfaction. In the dance field, self-kindness may be particularly important given the physical and emotional demands of the practice. Both professional and recreational dancers frequently encounter intense pressure to achieve technique, which can foster self-criticism and result in giving up. The findings suggest that self-compassion, particularly through the dimension of self-kindness, may buffer dancers against these challenges and contribute to improved life satisfaction. This is in line with research by Kosirnik et al. [32], who found that dancers who practiced self-compassion experienced greater emotional resilience and more positive experiences within their practices. Furthermore, a study by Zafeiroudi [12] has shown that self-compassion positively influences dancers’ emotional resilience and coping strategies, emphasizing the importance of self-kindness in dance environments.

The emphasis on self-kindness in this study lends strong support to the integration of Therapeutic-Based Art Pedagogy in recreational dance education. This pedagogical model reframes the role of the dance educator, not merely as an instructor of technique, but as a facilitator of emotional growth, self-compassion, and psychological well-being. Within this framework, dance becomes a site where movement and reflection co-exist, allowing students to engage in both expressive exploration and internal regulation. Such environments promote a non-judgmental, emotionally safe climate where participants are encouraged to accept personal imperfections and develop resilience. Especially in non-competitive, artistic, leisure-based settings, this approach may buffer dancers from the perfectionism and self-judgment often associated with more traditional or performance-driven teaching. As the findings suggest, reinforcing emotional safety and kindness within the learning environment may have significant effects on life satisfaction, positioning dance education as a form of applied psychology grounded in embodied experience [6,7].

### 4.2. Isolation and Life Satisfaction: The Role of Connection in Dance

The dimension of isolation emerged as the strongest among all self-compassion dimensions in the present study. Feelings of isolation, or the belief that one is fundamentally different from others, were strongly associated with lower life satisfaction. This finding aligns with existing research on self-compassion, which consistently shows that individuals who experience isolation or social disconnection tend to report poorer mental health outcomes [49]. In the dance domain, isolation may stem from the often-solitary nature of practice or the competitive environment inherent in many dance settings. Professional dancers frequently work long hours in isolation, striving to perfect their technique, and may feel alienated from their peers due to the physical and artistic demands of their craft. Studies in art-based settings such as theatre and professional sports have found similar patterns, where feelings of isolation can exacerbate stress, hinder social support networks, and negatively affect overall well-being [50]. Recreational dance settings often foster a more inclusive environment where people of all backgrounds and skill levels come together, while there may still be feelings of social isolation, according to dance style demands or teacher’s background and the teaching style.

In dance therapy settings, fostering connection through dance and expressive movement can reduce feelings of isolation and enhance social connectedness. Dance offers a platform for individuals to experience and express emotions, which is an antidote to the social isolation commonly associated with mental health issues [12,51]. The significant negative impact of isolation on life satisfaction corroborates extensive literature in both the self-compassion field and in dance education and dance therapy, highlighting the importance of addressing social disconnection to enhance well-being [52,53].

### 4.3. Self-Judgment and Life Satisfaction: The Negative Impact of Harsh Self-Criticism

Findings also revealed a significant negative relationship between self-judgment and life satisfaction. While statistically significant, the relative contribution of self-judgment was modest, suggesting a weaker unique predictive value in comparison to other components. This is consistent with previous research indicating that high levels of self-judgment, or the tendency to evaluate oneself harshly, are associated with lower well-being [13]. Self-judgment often results in feelings of inadequacy, self-doubt, and anxiety, which can undermine an individual’s sense of self-worth and overall life satisfaction. In the field of dance, where dancers are regularly subjected to critical feedback from instructors, peers, and audiences, a tendency toward highly self-critical may exacerbate these effects. This finding aligns with Atienza et al. [54], who reported that dancers with higher levels of self-judgment experienced greater levels of anxiety and lower psychological well-being. Interestingly, in the present study, self-judgment was not fully counterbalanced by the other dimensions of self-compassion. While self-kindness provides protection against self-criticism, it may be that dancers need specific interventions targeting self-judgment to reduce its negative effects. This emphasizes the importance of self-compassion training programs that focus on reducing harsh self-judgment and promoting a more compassionate self-view, particularly in technique-focused environments like dance.

### 4.4. Common Humanity and Life Satisfaction

The finding that common humanity exhibited a limited positive but marginally non-significant effect on life satisfaction provided insights into the role of self-compassion in dancers’ well-being. Common humanity, a core component of self-compassion, involves recognizing that personal struggles are part of a shared human experience [13]. This perspective may reduce feelings of isolation, fostering resilience and psychological well-being. However, the marginal significance in this study suggested a complex interaction between common humanity and the specific cultural and practice context.

Dancers operate in a unique environment characterized by collective practices, group performances, and a shared pursuit of artistic goals. These dynamics could influence the relationship between common humanity and well-being. Studies in the context of dance provide mixed insights. Quested and Duda [55] found that perceptions of social support and relatedness significantly predicted motivation and well-being in dancers. These findings suggest that dancers may already benefit from an inherent sense of communal identity, potentially diluting the independent effect of common humanity on life satisfaction. Other studies highlighted that in collectivist cultures, such as Greece, shared cultural practices like dance promote natural group bonding and collective resilience [56,57]. This cultural predisposition may reduce the distinct impact of common humanity.

An explanation for this study’s finding could be the pre-existing connectedness. Dance inherently fosters a sense of shared experience and interdependence through group rehearsals and performances. This baseline connectedness might mask the additional contribution of common humanity to life satisfaction. Some other explanations could be the cultural context and the performance focus. In Greece, where collectivism and communal activities are deeply ingrained, dancers may already internalize a sense of shared human experience, rendering the explicit recognition of common humanity less distinct as a predictor [58]. Furthermore, unlike non-dancers, who might benefit from common humanity as a means of reducing self-critical tendencies, dancers may focus more on technical and performance-related aspects of their identity, shifting the relevance of self-compassion components. While common humanity may be less salient in contexts where communal values are already emphasized, it could play a critical role in settings where dancers face heightened isolation, such as competitive environments or solo-focused dance styles.

### 4.5. Mindfulness and Life Satisfaction: A Marginal Role

Despite the well-documented benefits of mindfulness in promoting psychological well-being [59,60,61,62], this study did not find a significant relationship between mindfulness and life satisfaction. This finding contrasts with some studies that have shown mindfulness to be a key predictor of improved well-being and reduced emotional distress [63,64]. One potential explanation for this difference is that dancers may naturally develop high mindfulness levels through their practice. This pre-existing mindfulness could reduce variability within the sample, making it harder to detect a direct relationship with life satisfaction. Mindfulness might also indirectly influence life satisfaction through other variables, such as emotional resilience or self-compassion components like self-kindness, rather than having a direct effect. Another possibility is that the sample of dancers in this study may have already been relatively high in mindfulness, making it harder to detect additional effects. Previous research on elite athletes and performers suggests that individuals in these domains may already exhibit high levels of mindfulness due to the nature of their training and practice, which could reduce the variability in mindfulness scores needed to detect significant effects on life satisfaction [65,66]. Another reason could be that in recreational dance, where enjoyment, group harmony, and shared experiences are emphasized, the individual benefits of mindfulness may be less significant compared to other self-compassion dimensions like self-kindness or common humanity.

### 4.6. Psychological and Emotional Impacts of Dance Styles

The findings revealed statistically significant but small effects of dance styles on certain psychological variables. Specifically, participants in tango reported the highest levels of common humanity, reflecting the style’s emphasis on relational dynamics and partner connection, which likely fosters a stronger sense of shared human experience. Small yet significant differences were also observed for self-kindness and self-judgment, with Latin-American and tango dancers scoring higher in self-kindness but also reporting slightly elevated self-judgment. This suggests that these styles may cultivate both positive self-regard and critical self-reflection. Regarding mindfulness, tango dancers demonstrated higher levels compared to participants in dance aerobic, possibly due to the style’s intricate, slow-paced movements that require greater embodied attention. However, life satisfaction and isolation did not differ significantly across styles, suggesting that recreational dance broadly contributes to well-being and social connection regardless of genre.

While the observed differences across styles were modest in practical significance, they point to potential trends worth further exploration. Partner-based or emotionally expressive dance forms (Tango and Latin) may encourage particular psychological traits, whereas more individual-focused styles, such as ballet, have been associated with higher self-critical tendencies [67,68]. These findings indicate that, although all dance forms contribute positively to emotional well-being, some may offer unique psychological benefits depending on their structure and expressive demands.

Beyond these style-based differences, this study also revealed broader psychological patterns across the full sample of recreational dancers. These findings align with the principles and therapeutic benefits of dance movement, emphasizing the interrelation of physical movement, emotional resilience, and life satisfaction. The predictive role of self-compassion, particularly self-kindness, mirrors the core objectives of dance movement therapy, where fostering a nurturing relationship with oneself is essential. In dance movement therapy, self-kindness is developed through expressive and nonjudgmental movement, enabling individuals to process emotions and reduce harsh self-criticism. This study’s identification of isolation as a significant negative predictor of life satisfaction further supports the dance movement therapy framework, which actively addresses social disconnection through group-based movement activities [1]. By fostering shared emotional and physical experiences, dance movement promotes a sense of common humanity and reduces feelings of isolation, mirroring findings that social connection plays a key role in well-being.

Moreover, the absence of a significant effect of mindfulness on life satisfaction in this study underlines the potential for dance movement therapy to serve as a complementary intervention. While mindfulness practices focus on present-moment awareness, dance movement therapy integrates this awareness with embodied cognition, enhancing the therapeutic impact for individuals who may already possess high baseline levels of mindfulness, as seen in dancers [1,5]. The study’s focus on recreational dance, with its emphasis on emotional engagement rather than performance pressure, aligns with dance movement therapy’s adaptable approach to fostering holistic well-being. By addressing both psychological and social dimensions, dance movement in therapy offers a framework to expand upon the study’s findings, highlighting how integrative practices can further enhance life satisfaction and emotional health among recreational dancers [1,69].

These patterns show how recreational dance not only supports emotional regulation and social connection but also exemplifies the process of choreographing well-being, an embodied and relational approach to psychological growth that integrates movement, self-awareness, and interpersonal presence [6,7,9]. The findings confirm that dance functions as more than a leisure activity; it acts as a living practice where well-being is co-created through dynamic engagement with the body, others, and one’s emotional world.

### 4.7. Implications for Dance Practice and Future Research

This study offers significant practical implications for dance education, performance psychology, and psychotherapy, emphasizing the potential importance of self-compassion in fostering emotional resilience and social connection. These findings support the integration of Therapeutic-Based Art Pedagogy, a pedagogical framework that merges expressive movement with psychological support. This approach conceptualizes dance instruction not only as artistic or technical training but as an emotionally attuned, developmentally supportive process that promotes mental health and well-being.

Dance educators and choreographers could incorporate self-compassion training into their teaching practices to enhance both individual well-being and group cohesion. By promoting positive self-regard and minimizing perfectionism, instructors help dancers develop healthier attitudes toward themselves and their art. This could be operationalized through structured activities, such as guided improvisation, expressive journaling, and compassion-based warm-up sequences, which encouraged reflection and emotional safety within the classroom.

Creating a supportive environment that emphasizes emotional connection moderates the adverse effects of isolation and fosters a sense of belonging within dance communities. Within a Therapeutic-Based Art Pedagogy model, this environment was actively co-constructed through compassionate communication, collaborative learning, and group cohesion strategies. Kindness, directed toward oneself and others, appears influential in reducing harsh self-judgment and peer criticism. Educators could reinforce this by fostering a nurturing atmosphere that prioritizes kindness, constructive feedback, and mutual respect. This approach not only relieves technique-related stress but also strengthens group identity through shared experiences, such as collaboratively overcoming challenges in choreography.

The communal nature of dance, as highlighted by the strong correlation between self-kindness and common humanity, illustrates dance’s potential to promote mutual understanding and a sense of collective purpose. Mindfulness also emerged as a valuable tool for addressing performance-related stress and emotional dysregulation. Instructors and therapists could integrate mindfulness-based techniques, such as breathing exercises, body scanning, and guided visualization, to help dancers manage self-critical thoughts and cultivate embodied awareness.

In collectivist dance cultures, such as traditional Greek dance, where community and shared experience were central, the principles of Therapeutic-Based Art Pedagogy are often already embedded. Conversely, in individualistic or competitive dance settings, where perfectionism, isolation, and judgment are more prevalent, intentional efforts to incorporate kindness, emotional literacy, and mindfulness where are particularly beneficial in supporting dancers’ mental health and social connection. Dance therapy, in particular, provides a unique platform to explore both the adaptive and maladaptive dimensions of self-compassion. Group sessions allowed participants to connect through shared movement, promoting affective attunement and reducing feelings of isolation.

The integration of self-compassion training into both dance education and psychotherapy involves structured practices that address the psychological challenges dancers face. Reflective exercises, such as journaling prompts that encourage self-kindness and reframe self-critical thoughts, support the development of a healthier internal narrative. Mindfulness-based movement practices, including gentle stretching, somatic tracking, and guided embodiment, help dancers reconnect with their emotional and physical selves. Group activities, such as co-created movement phrases and collective improvisations, build a sense of common humanity that counters isolation and deepens emotional connection.

Therapists and educators play a crucial role in modeling self-compassion through empathetic presence and communication. By creating safe and responsive environments, they could empower dancers to navigate common challenges such as performance anxiety, physical vulnerability, and artistic self-doubt. Specialized workshops and restorative routines, such as compassion-focused warm-ups, somatic self-care rituals (Gyrotonic, yoga, or body awareness exercises), and movement-based emotional processing, offer practical strategies to enhance psychological resilience.

Beyond the dance setting, insights from this study inform interventions for broader populations, especially individuals struggling with perfectionism, isolation, or emotional suppression. Mindfulness, reflective journaling, and movement-based group activities emphasizing self-compassion could be adapted across psychotherapeutic contexts. Approaches such as Compassion-Focused Therapy (CFT) might be enriched by integrating movement as a means of embodying and deepening emotional healing. These strategies highlight the universal applicability of Therapeutic-Based Art Pedagogy principles in promoting resilience, self-regulation, and sustainable well-being through the arts.

### 4.8. Limitations and Future Research

While this study provided valuable insights, its design limits causal inferences. The use of self-report measures may introduce biases such as social desirability or subjective distortion. While the scales showed strong psychometric properties, future studies could enhance validity by incorporating behavioral or observational data. Given the cross-sectional and correlational nature of the design, the results should be interpreted as statistical associations rather than causal relationships. The mindfulness measure had limitations, including potentially high baseline levels in dancers and a mismatch with life satisfaction. Addressing these through alternative measures or more specific contexts could better capture the relationship between mindfulness and life satisfaction in art-based populations. Additionally, exploring cultural differences may reveal how socio-cultural factors shape self-compassion, improving applicability. Future studies should focus on self-compassion training for self-judgment and isolation and assess whether dance-specific mindfulness practices have a greater impact on well-being.

## 5. Conclusions

This study contributes to the literature by highlighting the predictive role of self-compassion in dancers’ life satisfaction. The validation of the five-factor model of self-compassion supports its applicability and relevance within recreational dance settings. Self-kindness emerged as an important factor in fostering emotional resilience, while isolation and self-judgment negatively impacted life satisfaction. The findings also revealed that different dance styles influenced the expression of self-compassion dimensions, underscoring the importance of contextual and stylistic factors in dancers’ psychological experiences. These results suggested that dance educators and therapists may consider integrating self-compassion training into their programs, adapting practices to meet the unique psychological needs associated with different dance forms. In this context, Therapeutic-Based Art Pedagogy may provide an effective framework for aligning emotional development with artistic expression. Such an approach emphasizes embodied awareness, emotional safety, and social connection, core principles that are especially relevant in recreational dance. Dance movement therapy, with its focus on emotional expression, body–mind integration, and relational dynamics, could further complement these pedagogical goals. Structured programs grounded in this combined therapeutic-artistic perspective may help mitigate the negative effects of performance-related stress and isolation while simultaneously promoting mental health and group cohesion. Βy addressing both the technical and psychological dimensions of dance, educators and therapists have the potential to reframe dance practice into a medium for holistic development, one that nurtures not only creativity and physical skill but also emotional resilience, self-compassion, and long-term well-being.

## Figures and Tables

**Table 1:** Demographic and dance participation characteristics of the sample (*n* = 912).

Sex	Age	Income	Years of Participation	Dance Style
Men 20%	18–29 years 45.2%	Low 34.3%	≤1 year 11.1%	Ballet 15.8%
Women 80%	30–39 years 30.5%	Medium 60.5%	2–4 years 16.1%	Contemporary 24.5%
	40–49 years 16.8%	High 5.2%	5–7 years 14.4%	Traditional 19.5%
	50< years 7.6%		8 ≤ years 58.4%	LatinAmerican 13.5% Tango 10.7%
				Aerial 8%
				Dance aerobic 8%

**Table 2:** Descriptive statistics for self-compassion dimensions and life satisfaction (*n* = 912).

Factors	*M*	*S.D.*	Skewness	Kurtosis
Self-kindness	3.34	0.81	−0.17	−0.36
Mindfulness	3.45	0.80	−0. 29	−0.43
Common Humanity	3.04	0.86	−0.05	−0.63
Self-judgment	3.23	0.81	−0.29	−0.40
Isolation	3.58	0.89	−0.45	−0.40
Life satisfaction	4.69	0.61	−0.27	−0.27
Self-compassion	3.03	0.60	−0.29	−0.08

**Table 3:** Model fit indices.

Fit Index	Value	Recommended Threshold
CFI (Comparative Fit Index)	0.91	≥0.90
RMSEA (Root Mean Square Error of Approximation)	0.05	≤0.08
SRMR (Standardized Root Mean Square Residual)	0.05	≤0.08
TLI (Tucker–Lewis Index)	0.90	≥0.90
*χ*^2^/*df* (Chi-square/df)	3.1	≤3.0

**Table 4:** Standardized factor loadings for CFA.

Self-Compassion Dimension	Factor Loading (β)	Standard Error	Critical Ratio	Cronbach Alpha	CR	AVE
Self-Kindness				0.78	0.72	0.54
Item 12	0.673	0.09	14.09			
Item 5	0.539	0.08	12.09			
Item 23	0.558	0.07	15.12			
Mindfulness				0.76	0.78	0.61
Item 20	0.473	0.06	12.44			
Item 24	0.530	0.07	11.67			
Item 2	0.581	0.08	12.53			
Common Humanity				0.81	0.75	0.57
Item 1	0.457	0.09	10.33			
Item 11	0.579	0.09	12.01			
Item 21	0.509	0.08	11.16			
Self-judgment				0.78	0.71	0.53
Item 16	0.711	0.12	13.32			
Item 8	0.575	0.10	11.96			
Item 7	0.456	0.06	10.81			
Isolation				0.79	0.73	0.55
Item 25	0.663	0.06	12.98			
Item 13	0.625	0.06	15.43			
Item 4	0.605	0.05	15.01			

**Table 5:** Correlations between latent factors of the Self-Compassion Scale.

Factors	Self-Kindness	Self-Judgment	Common Humanity	Isolation	Mindfulness
Self-Kindness	1.00	0.694	0.798	0.665	0.734
Self-Judgment	0.694	1.00	0.356	0.821	0.785
Common Humanity	0.798	0.356	1.00	0.525	0.520
Isolation	0.665	0.821	0.525	1.00	0.913
Mindfulness	0.734	0.785	0.520	0.913	1.00

Note: All Pearson correlations were statistically significant at *p* < 0.001 (1-tailed).

**Table 6:** Regression analysis.

Dependent Variable Life Satisfaction					
Predictor Variable	Standardized Coefficients (β)	*t*-Value	*p*-Value	Structure Coefficients (r)	Pratt Index
Self-Kindness	0.258	6.241	<0.001	0.385	0.099
Self-judgment	−0.083	−2.192	0.029	0.223	−0.019
Common Humanity	0.064	1.842	0.066	0.234	0.015
Isolation	0.307	8.199	<0.001	0.389	0.119
Mindfulness	0.004	0.117	0.907	0.266	0.001

**Table 7:** Descriptive statistics for each dependent variable by dance style (*n* = 912).

Variables	*n*	Common Humanity	Self-Kindness	Self-Judgment	Isolation	Mindfulness	Life Satisfaction
Dance Styles		M/SD	M/SD	M/SD	M/SD	M/SD	M/SD
Ballet	144	2.86/0.83	3.17/0.77	3.10/0.80	3.54/0.93	3.30/0.72	4.75/1.2
Contemporary	223	3.00/0.92	3.42/0.78	3.15/0.87	3.49/0.88	3.46/0.79	4.74/0.99
Traditional	178	3.01/0.81	3.21/0.74	3.18/0.73	3.58/0.94	3.42/0.79	4.59/0.98
LatinAmerican	123	3.16/0.80	3.51/0.84	3.41/0.78	3.75/0.83	3.59/0.72	4.67/1.1
Tango	78	3.27/0.81	3.45/0.81	3.46/0.74	3.64/0.88	3.67/0.75	4.55/0.98
Aerial	73	3.08/0.88	3.35/0.97	3.26/0.77	3.58/0.84	3.58/0.92	4.82/1.8
Dance Aerobic	73	3.09/0.90	3.32/0.78	3.20/0.85	3.58/0.87	3.14/0.94	4.81/0.84

**Table 8:** Post-hoc comparisons between dance styles (Scheffé test).

Comparison Pair	Dependent Variable	Mean Difference	*p*-Value (Scheffé)	95% Confidence Interval	Cohen’s d
Tango vs. Classical Ballet	Common Humanity	0.543	<0.001	[0.25, 0.83]	0.50
Tango vs. Dance Aerobic	Common Humanity	0.508	<0.001	[0.21, 0.80]	0.21
Tango vs. Latin	Common Humanity	0.438	0.003	[0.14, 0.74]	0.13
Tango vs. Traditional	Common Humanity	0.356	0.028	[0.03, 0.68]	0.32
Tango vs. Classical Ballet	Mindfulness	0.378	0.004	[0.12, 0.64]	0.50
Tango vs. Dance Aerobic	Mindfulness	0.421	0.003	[0.13, 0.71]	0.63
Tango vs. Latin	Mindfulness	0.335	0.031	[0.01, 0.65]	0.11

Note: Effect-size: *d* ≈ 0.20 small, 0.50 medium, 0.80 large [46].

## Data Availability

The data presented in this study are available upon request from the corresponding author, due to ethical and privacy reasons, and will be shared in an anonymized format upon reasonable request.

## Abbreviations

The following abbreviations are used in this manuscript:

CFA	Confirmatory Factor Analysis
CFI	Comparative Fit Index
CR	Composite Reliability
DPESS	Department of Physical Education and Sport Sciences
IEC	Internal Ethics Committee
RMSEA	Root Mean Square Error of Approximation
SCS	Self-Compassion Scale
SRMR	Standardized Root Mean Square Residual
SWLS	Satisfaction With Life Scale
TLI	Tucker–Lewis Index
